# Abundance estimates and habitat preferences of bottlenose dolphins reveal the importance of two gulfs in South Australia

**DOI:** 10.1038/s41598-019-44310-3

**Published:** 2019-05-29

**Authors:** Kerstin Bilgmann, Guido J. Parra, Lauren Holmes, Katharina J. Peters, Ian D. Jonsen, Luciana M. Möller

**Affiliations:** 1 Cetacean Ecology, Behaviour and Evolution Laboratory (CEBEL), College of Science and Engineering, Flinders University, GPO Box 2100, Adelaide, South Australia 5001 Australia; 2 Molecular Ecology Laboratory, College of Science and Engineering, Flinders University, GPO Box 2100, Adelaide, South Australia 5001 Australia; 3 Marine Vertebrate Conservation and Evolution Laboratory, Department of Biological Sciences, Faculty of Science and Engineering, Macquarie University, North Ryde, Sydney, NSW 2109 Australia; 4 Global Ecology, College of Science and Engineering, Flinders University, GPO Box 2100, Adelaide, South Australia 5001 Australia; 5 Coastal-Marine Research Group, School of Natural and Computational Sciences, Massey University, Auckland, 0745 New Zealand

**Keywords:** Behavioural ecology, Conservation biology

## Abstract

Informed conservation management of marine mammals requires an understanding of population size and habitat preferences. In Australia, such data are needed for the assessment and mitigation of anthropogenic impacts, including fisheries interactions, coastal zone developments, oil and gas exploration and mining activities. Here, we present large-scale estimates of abundance, density and habitat preferences of southern Australian bottlenose dolphins (*Tursiops* sp.) over an area of 42,438km^2^ within two gulfs of South Australia. Using double-observer platform aerial surveys over four strata and mark-recapture distance sampling analyses, we estimated 3,493 (CV = 0.21; 95%CI = 2,327-5,244) dolphins in summer/autumn, and 3,213 (CV = 0.20; 95%CI = 2,151-4,801) in winter/spring of 2011. Bottlenose dolphin abundance and density was higher in gulf waters across both seasons (0.09-0.24 dolphins/km^2^) compared to adjacent shelf waters (0.004–0.04 dolphins/km^2^). The high densities of bottlenose dolphins in the two gulfs highlight the importance of these gulfs as a habitat for the species. Habitat modelling associated bottlenose dolphins with shallow waters, flat seafloor topography, and higher sea surface temperatures (SSTs) in summer/autumn and lower SSTs in winter/spring. Spatial predictions showed high dolphin densities in northern and coastal gulf sections. Distributional data should inform management strategies, marine park planning and environmental assessments of potential anthropogenic threats to this protected species.

## Introduction

Globally, a quarter of all large mammal species are threatened with extinction and an additional 800+ species are classified as ‘data deficient’^1^. To determine the conservation status of particular populations or species, and to make appropriate management decisions, data on their abundance and distribution are needed, particularly for those species currently under threat^2^. Marine mammals are slow breeding, highly mobile marine predators, which are particularly vulnerable to anthropogenic impacts^1,3,4^. Most anthropogenic threats to small marine mammals such as dolphins occur in coastal areas that are heavily utilised by humans^5–7^. Thus, accurate assessments of population size and habitat preferences are needed to understand the dynamics of dolphin populations, and inform conservation and management decisions.

The distribution of coastal bottlenose dolphins (*Tursiops* spp.) is usually patchy and dependent on habitat type and availability of food resources^8–10^. Although coastal bottlenose dolphins are well studied at a global scale, there is a lack of abundance data for many regions. This makes it difficult to assess the level of threats dolphin populations may be exposed to and to make informed decisions about environmental impact assessments and marine parks planning^11,12^. In Australian waters, all dolphins are protected under the *Environment Protection and Biodiversity Conservation Act 1999* and by legislation pertaining to waters under the jurisdiction of each State (i.e. up to 3 nautical miles from shore). There is a deficiency of density and abundance data in Australian waters for dolphins over large geographical scales in the order of thousands of km^2^, and therefore such data have recently been of key interest for developing fisheries bycatch mitigation strategies^11^.

In South Australian waters, two putative bottlenose dolphin species occur. The common bottlenose dolphin (*Tursiops truncatus*) is mainly found in deeper shelf and offshore environments, but in some areas of Australia it also occurs close to the coast^13^. The recently described southern Australian bottlenose dolphin (also named Burrunan dolphin; *Tursiops australis*)^14^ occurs in coastal waters of southern Australia, including South Australia^14–16^. This species is currently not widely recognized as a separate species by the marine mammal scientific community^17,18^. Evidence exists that *T*. *australis* is genetically distinct from other bottlenose dolphin species^14,19–21^, but morphological evidence is currently insufficient to confirm this^14,22^. We use only the term ‘bottlenose dolphins’ to refer to dolphins studied here, likely to be putative species *T. australis* due to its proximity to the coast and small school sizes.

**Figure 1:**
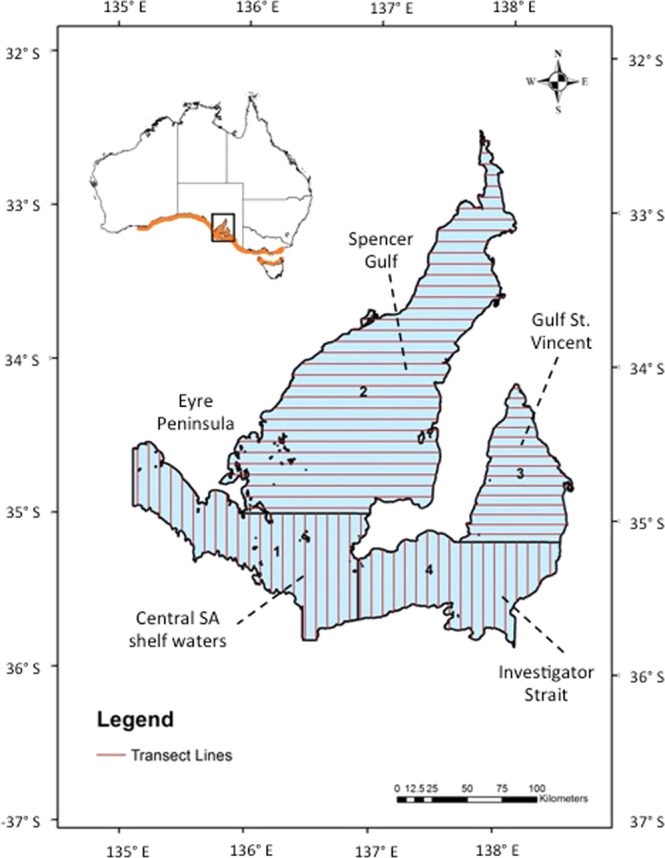
Map of the study area in South Australia (SA) with line-transect survey layout, showing the division of the area into four strata: (1) South Australian shelf waters; (2) Spencer Gulf; (3) Gulf St Vincent; and (4) Investigator Strait. The equally spaced continuous lines in dark grey/red indicate the line-transect layout approximately perpendicular to the coast. Black lines indicate the coastline, the divisions between strata, and for the southern section of stratum 1 the 100 m depth contour of the Australian continental shelf. The approximate distribution of the coastal bottlenose dolphin (putative *Tursiops australis*) along the southern Australian coast is displayed in grey/orange in the overview of Australia. Along the marked grey/orange area, the distribution of dolphins does not appear to be continuous, but rather may focus in gulf and embayment waters, and to some coastal beaches.

The lack of information for bottlenose dolphins, including abundance, distribution and habitat preferences, makes it difficult to assess vulnerability to anthropogenic threats. A recent study revealed the existence of hierarchical metapopulation genetic structure for bottlenose dolphins in most of southern Australia^16^. Two dolphin populations were identified in the two major South Australian gulfs, Spencer Gulf and Gulf St Vincent (Fig. 1), and these dolphins showed restricted gene flow to dolphins outside the gulfs^16^. Bottlenose dolphin abundance estimates for the two genetic populations of the South Australian gulfs are therefore particularly desirable to inform conservation management and to facilitate parameters for population modeling and viability analyses. Based on previous population genetic studies of coastal bottlenose dolphins in southern Australian waters, a total of six separate genetic populations of the same species have so far been identified^15,16,23^. Some of these populations inhabit small embayments and may be particularly vulnerable to human-induced threats^14,24–27^. In waters of the southern Australian state of Victoria, for example, two genetically distinct populations^15^, are currently listed as threatened under the *Victorian Flora and Fauna Guarantee Act 1988*.

Known threats to coastal dolphins in South Australian waters include bioaccumulation of heavy metals^28,29^, epizootic events^30^, habitat destruction and/or displacement, coastal development, risk of boat strikes, increased noise pollution, climate change^12^, intentional killings^31^ and fisheries by-catch^31–35^. In southern Australia, known areas of high use by bottlenose dolphins that are also heavily used by humans are (1) the metropolitan coastal waters of Adelaide, the capital city of South Australia with a population of approximately 1.3 million people; and (2) Port Phillip Bay located off Melbourne, the capital city of Victoria, with a population of approximately 4.4 million people. Both urban areas are known for their frequent boat traffic and ongoing coastal development^24–26,36,37^. An anthropogenic threat assessment in Spencer Gulf, South Australia, rated climate change (temperature and storms, ocean warming, ocean acidification and salinity increase) and noise disturbance as the most prominent current threats to bottlenose dolphins in the area, followed by haul and gillnet fisheries^12^.

In South Australian coastal waters, bottlenose dolphin abundance has been estimated in several small-scale regional areas using boat-based, photo-identification surveys including metropolitan Adelaide, Port River estuary and Barker Inlet in Gulf St Vincent, and Coffin Bay^26,38,39^. However, large-scale estimates of abundance, density and habitat preferences are lacking. Available data cannot be used to extrapolate abundance and densities of bottlenose dolphins to other geographic regions given the inherent heterogeneity of environmental conditions across such large scales and the technical and fundamental challenges in the transferability of ecological models^40^. Thus, implementing surveys over large areas that have not yet been surveyed is central to understanding habitat preferences and identifying areas of high dolphin occurrence that can inform regional conservation management.

Here we present estimates of abundance and habitat preferences of bottlenose dolphins from large-scale aerial surveys undertaken in two South Australian gulfs, the predominant region in which anthropogenic interactions with delphinids occur^11,12,31,41,42^. The objectives of our study were to: (1) estimate the abundance of bottlenose dolphins for two distinct genetic populations, one in each South Australian gulf, and in the adjacent coastal and shelf waters; (2) identify areas of high dolphin density for summer/autumn and winter/spring seasons; and (3) investigate which environmental variables and habitat features correlate with high dolphin occurrence in the region. Our results provide valuable biological information on bottlenose dolphin abundance and habitat requirements that should aid marine park planning and environmental impact assessments of potential anthropogenic threats affecting this species.

## Results

**Figure 2:**
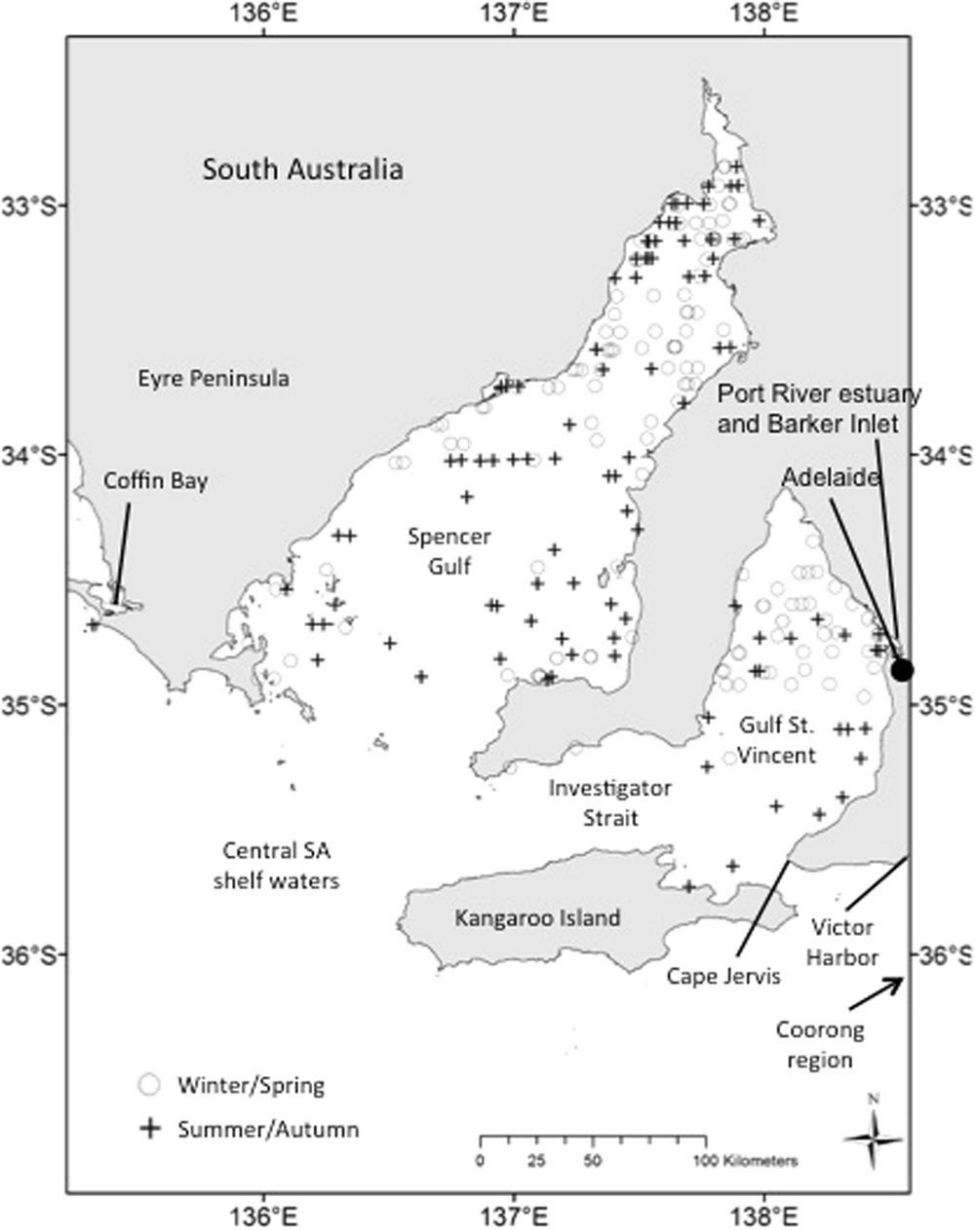
Distribution of coastal bottlenose dolphins recorded during aerial line-transect surveys in central South Australia (SA), using double observer platforms. Summer/autumn sightings are displayed as black crosses, and winter/spring sightings as non-filled circles. All unique bottlenose dolphin sightings on transect were included (no truncation of data).

**Figure 3:**
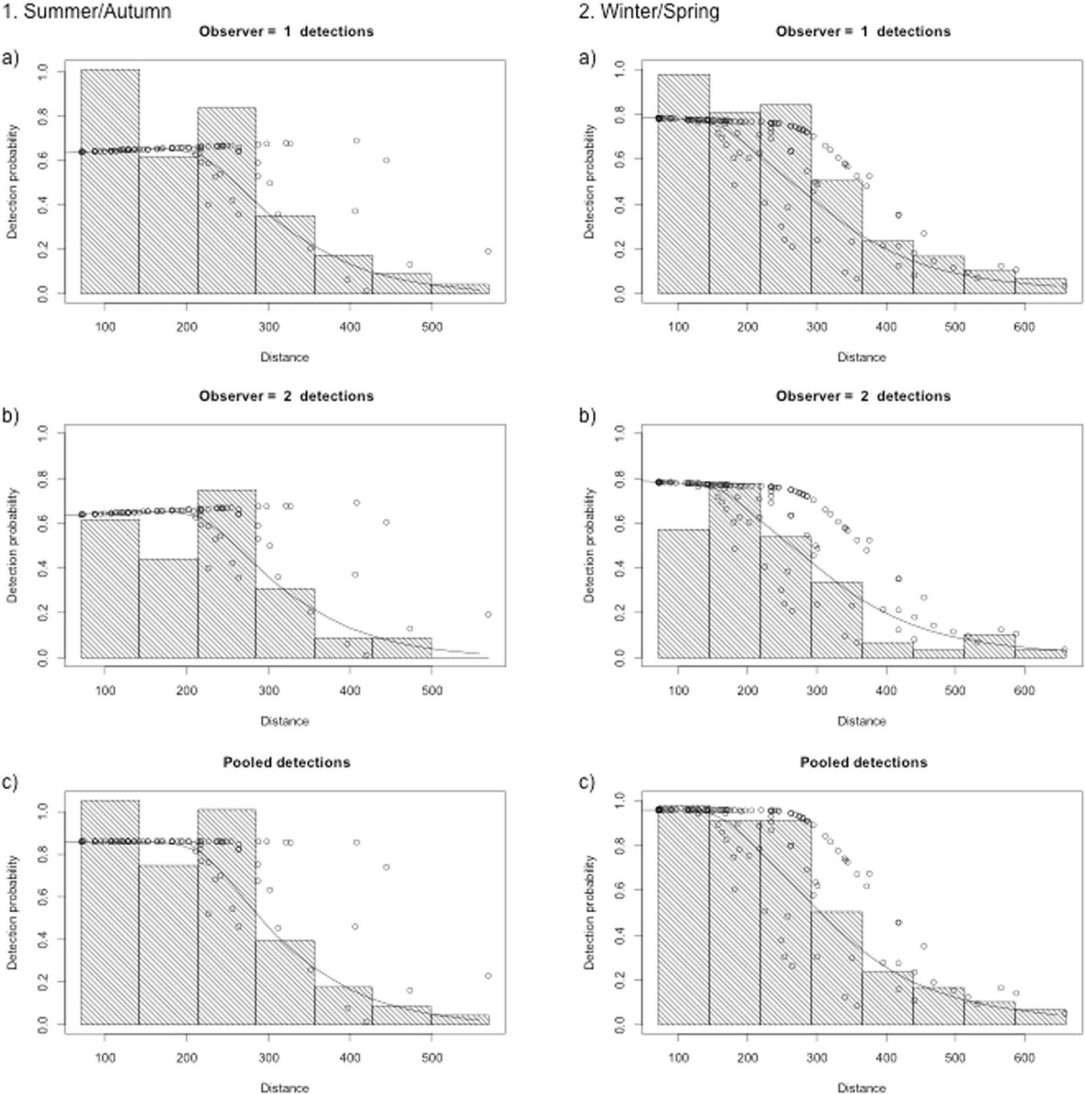
1. Detection function plots for systematic line-transect surveys flown in central South Australian in summer/autumn 2011. (**a**) Observer 1 detections (mid-seats); (**b**) Observer 2 detections (rear-seats); and (**c**) Pooled detections. Data were left truncated at 71 m (to adjust for obstructed view to the transect line) and right truncated at 570 m (to remove outliers at long distances); 2. Detection function plots for systematic line-transect surveys flown in central South Australian in winter/spring 2011. (**a**) Observer 1 detections; (**b**) Observer 2 detections; and (**c**) Pooled detection. Data were left truncated at 71 m (to adjust for obstructed view to the transect line) and right truncated at 660 m (to remove outliers at long distances).

A total of 5,198.0 km of transect line were flown during the summer/autumn survey and 5,235.8 km during the winter/spring survey (Table 1; Fig. 1). During the summer/autumn survey, we recorded 80 unique sightings of bottlenose dolphins, and 115 unique sightings during the winter/spring survey (Table 1; Figs 2 and 3).

**Table 1:** Summary of aerial surveys carried out in central South Australia (SA) by season for each stratum and overall: size of areas (km^2^), survey effort (km), number of bottlenose dolphins (*Tursiops* spp.; likely *T. australis*) school sightings made by each observer platform (after truncation) and number of unique school sightings (after truncation).

Stratum	Area (km^2^)	Effort (km)	Start and end date (day/month/year)	Number of transects	Number of sightings observer 1	Number of sightings observer 2	Number of unique sightings
***Summer/Autumn survey***							
1	9,042.2	1,085.7	17/04–06/06/2011	21	0	1	1
2	21,026.8	2,593.7	05/04–30/05/2011	30	57	39	59
3	5,103.6	636.3	27/03–29/03/2011	15	8	8	13
4	7,265.2	882.2	29/03–06/04/2011	18	6	6	7
**Total**	**42,437.8**	**5,198.0**	27/03–06/06/2011	**84**	**71**	**52**	**80**
***Winter/Spring survey***							
1	9,042.2	1,100.3	30/08–16/09/2011	21	0	0	0
2	21,026.8	2,613.7	16/09–05/10/2011	32	71	45	77
3	5,103.6	693.2	13/08–18/09/2011	16	33	25	36
4	7,265.2	828.6	09/08–13/08/2011	17	2	2	2
**Total**	**42,437.8**	**5,235.8**	09/08–05/10/2011	**86**	**110**	**73**	**115**

### Abundance, density and school sizes

For the summer/autumn survey, the mark-recapture distance sampling (MRDS) model with best fit was a distance sampling (DS) model with a hazard rate key function scaled with the covariates Beaufort sea state and cloud cover; and a mark-recapture (MR) model specified with perpendicular distance (Supplementary Table S1a). For the winter/spring survey, the MRDS model with best fit was a DS model with a hazard rate key function scaled with the covariates glare and cloud cover; and a MR model specified with perpendicular distance (Supplementary Table S1b). The addition of any other combination of covariates either produced the same or higher Akaike Information Criterion (AIC) values (Supplementary Tables S1a and S1b). The estimated abundance of bottlenose dolphins over the whole study area was 3,493 (CV = 0.21; 95 % CI = 2,327–5,244) for the summer/autumn survey, and 3,213 (CV = 0.21; 95 % CI = 2151–4801) for the winter/spring survey. Abundance estimates varied considerably between strata, revealing higher dolphin numbers in the two South Australian gulfs compared to shelf waters (Table 2). In both seasons, highest bottlenose dolphin density (dolphin/km^2^) was estimated for Gulf St Vincent (summer/autumn survey: D = 0.14; 95 % CI = 0.06–0.31; and winter/spring survey: D = 0.24; 95 % CI = 0.13–0.43). Second highest bottlenose dolphin density (dolphin/km^2^) was estimated for Spencer Gulf followed by Investigator Strait (Table 2). The lowest bottlenose dolphin density was estimated for central South Australian shelf waters, with very low numbers for the winter/spring survey and no dolphins observed in shelf waters during the summer/autumn survey (Table 2). Overall, dolphin school sizes ranged between one and 20 animals, with a mean school size of 2.70 (CV = 0.11) during the summer/autumn survey, and 2.03 (CV = 0.08) during the winter/spring survey (Table 3). While in Gulf St Vincent mean school sizes differed little between the two seasons, dolphins were seen in slightly smaller schools in Spencer Gulf and Investigator Strait during winter/spring, but this difference was not statistically significant (Table 3).

**Table 2:** Seasonal estimates of bottlenose dolphin (*Tursiops* spp.; likely *T. australis*) abundance with coefficient of variance (CV) and 95% confidence interval (CI), and bottlenose dolphin density (animals/km^2^) with 95% CI. Estimates are given for each stratum/region, and for the study area overall.

Stratum	Region	Animal abundance	CV	95% confidence interval	Density (animals/km^2^)	95% confidence interval
***Summer/Autumn survey***						
1	Central SA shelf waters	39	1.02	7–228	0.004	0.001–0.03
2	Spencer Gulf	2,431	0.23	1,530–3,862	0.12	0.07–0.18
3	Gulf St Vincent	708	0.40	318–1576	0.14	0.06–0.31
4	Investigator Strait	315	0.47	125–790	0.04	0.02–0.11
**Total**	**Study area overall**	**3,493**	**0.21**	**2,327–5,244**	**0.08**	**0.06–0.12**
***Winter/Spring survey***						
1	Central SA shelf waters	0	0.00	0	0.00	0.000
2	Spencer Gulf	1,952	0.26	1,169–3,260	0.09	0.06–016
3	Gulf St Vincent	1,202	0.30	657–2,201	0.24	0.13–0.43
4	Investigator Strait	59	0.77	14–241	0.01	0.002–0.03
**Total**	**Study area overall**	**3,213**	**0.20**	**2,151–4,801**	**0.08**	**0.05–0.11**

**Table 3:** Seasonal estimates of bottlenose dolphin (*Tursiops* spp.; likely *T. australis*) number of schools with coefficient of variance (CV) and 95% confidence interval, and mean school size with CV. Estimates are given for each stratum/region, and for the study area overall.

Stratum	Region	Number of schools	CV	95% confidence interval	Mean school size	CV
***Summer/Autumn survey***						
1	Central SA shelf waters	20	1.02	3–114	2.00	0.00
2	Spencer Gulf	894	0.22	582–1,375	2.72	0.15
3	Gulf St Vincent	266	0.36	128–552	2.66	0.09
4	Investigator Strait	114	0.35	56–229	2.77	0.29
**Total**	**Study area overall**	**1,294**	**0.19**	**885–1,892**	**2.70**	**0.11**
***Winter/Spring survey***						
1	Central SA shelf waters	0	0.00	0	0.00	0.00
2	Spencer Gulf	1,075	0.24	673–1,717	1.82	0.09
3	Gulf St Vincent	469	0.25	282–782	2.56	0.17
4	Investigator Strait	38	0.71	10–145	1.53	0.24
**Total**	**Study area overall**	**1,582**	**0.19**	**1,088–2,302**	**2.03**	**0.08**

Bottlenose dolphins sighted in this study were likely to be the putative Burrunan dolphin species (*T. australis*) based on their coastal distribution and smaller school sizes, compared to the common bottlenose dolphin (*T. truncatus*)^13–15,23,43,44^. The common bottlenose dolphin (*T. truncatus*), although previously recorded in offshore waters in the wider region, was likely not seen during either of the seasonal surveys, although genetic samples would be needed to confirm this.

### Habitat associations and spatial predictions

**Figure 4:**
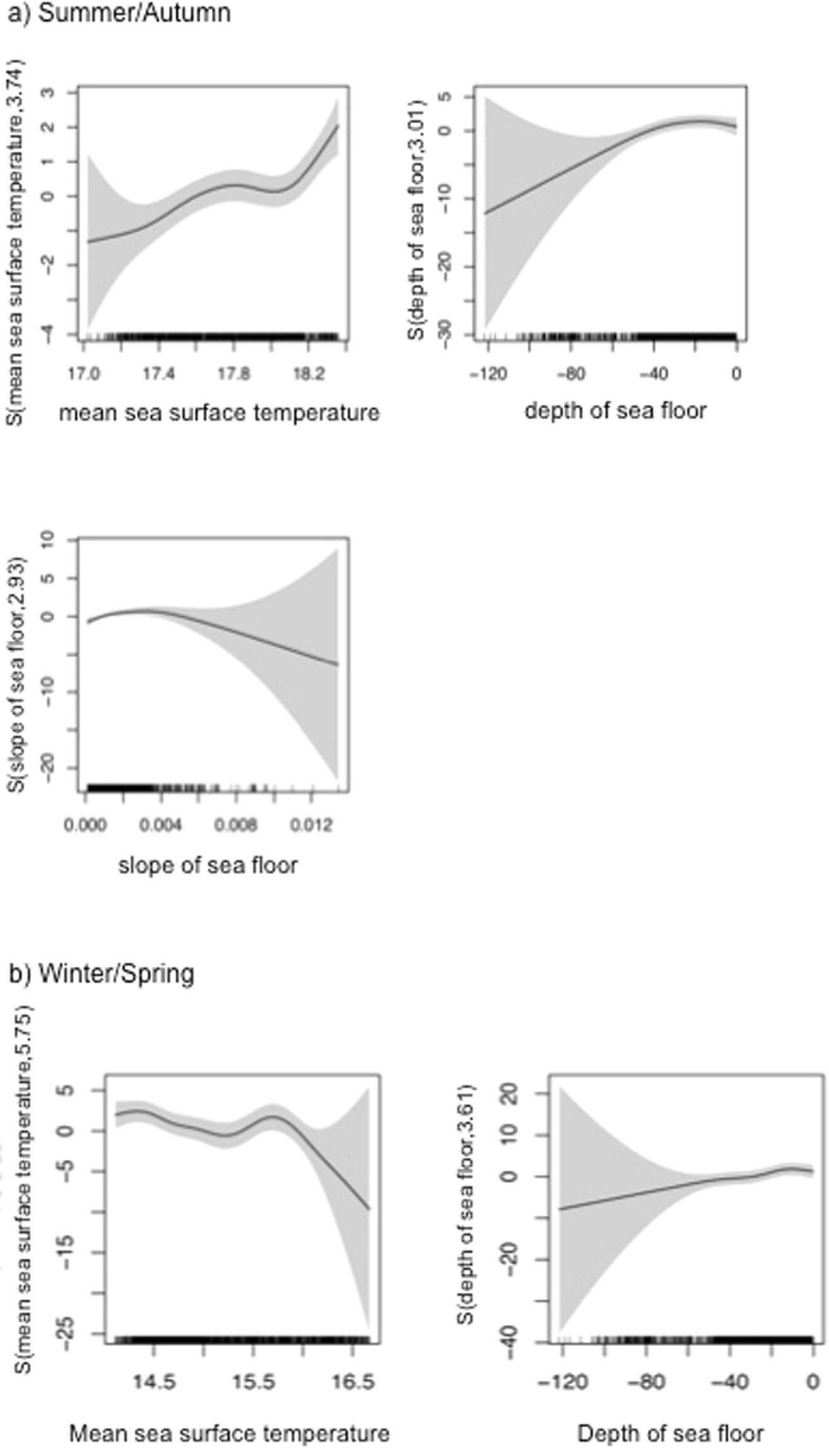
Plots of GAM smooth terms for the reduced models fit separately to summer/autumn and winter/spring aerial line-transect surveys. Predictor variables in the reduced (final) models included: mean sea surface temperature during the summer/autumn survey; mean sea surface temperature during the winter/spring survey; depth of sea floor; and slope of sea floor. Each term’s spline basis function with estimated degrees of freedom is displayed on the y-axes. The y-axis scales differ among terms to emphasize model fit. Shaded regions display 2x the standard error of the estimated smooth function. Vertical ticks on the x-axes denote the distribution of the data. The GAM for the summer/autumn survey explained 25.7% of the deviance, and the one for the winter/spring survey 34.1%.

For the summer/autumn survey, the Generalised Additive Models (GAMs) with the best fit included the predictor variables bathymetry, bathymetry gradient and mean sea surface temperature (SST), and explained 25.7% of the deviance. For the winter/spring survey, the GAM with best fit included bathymetry and mean SST, explaining 34.1% of the deviance. The GAMs revealed that bottlenose dolphins in the summer/autumn season were associated with shallow waters and a flat seafloor topography, and had a preference to warm SSTs of around 18–18.4 °C (Fig. 4a). In the winter/spring season, bottlenose dolphins were also associated with shallow waters, but had a preference to cooler mean SSTs of around 14–16 °C (Fig. 4b).

**Figure 5:**
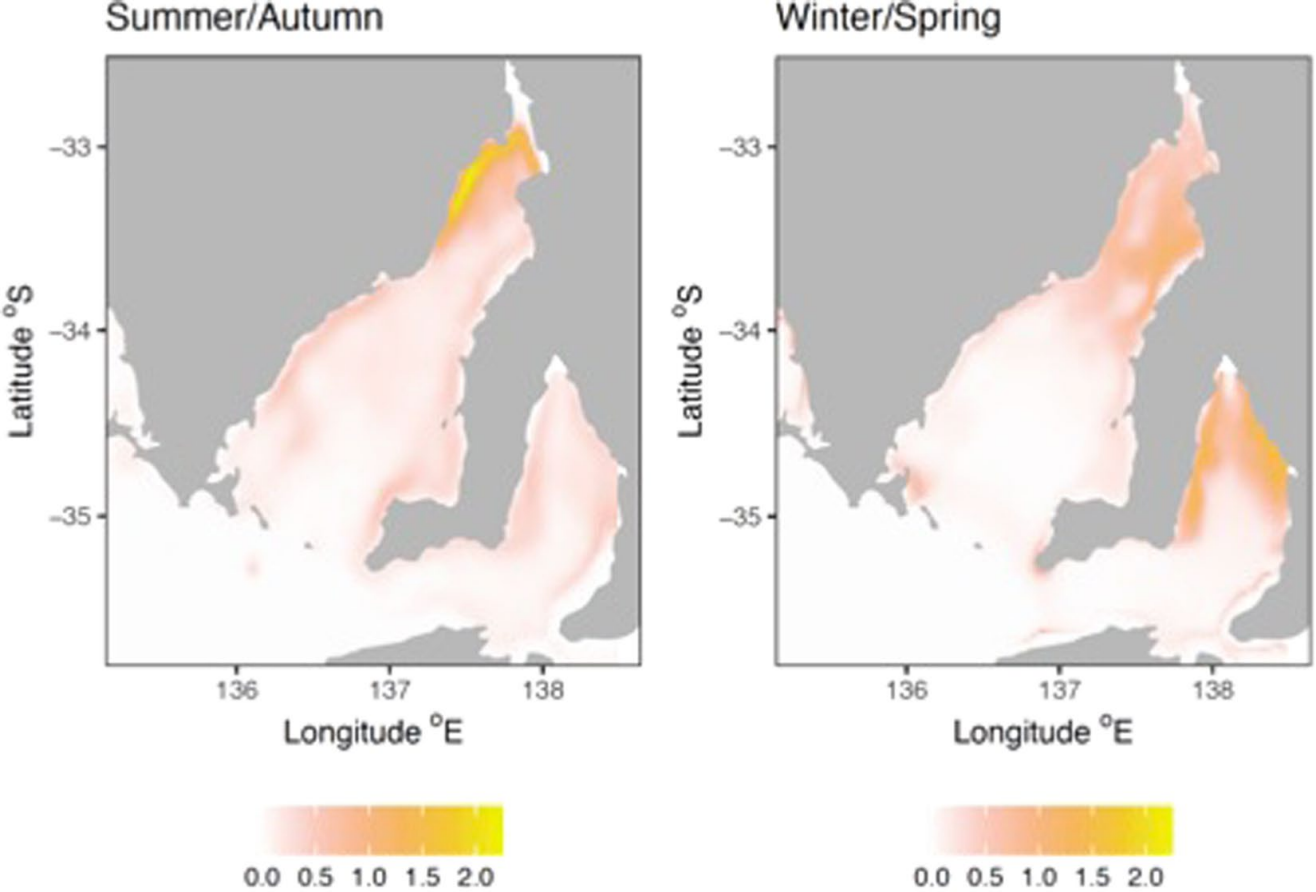
Spatial predictions of southern Australian bottlenose dolphin abundance according to the best fitting GAMs, displayed as predicted number of dolphins per 37 km^2^.

Spatial predictions from these models for both seasons revealed generally higher densities of dolphins in both gulfs, particularly in shallow and coastal waters, while central deeper gulf and shelf waters had lower predicted densities (Fig. 5). The highest densities of dolphins overall were predicted for both upper gulf regions especially in the winter/spring season for upper Gulf St Vincent. The waters of upper Gulf St Vincent with highest predicted dolphin densities included the heavily urbanized Adelaide metropolitan area, located on the east coast of Gulf St Vincent (Fig. 5).

## Discussion

Due to the number of potential threats faced by dolphins in central South Australia, estimates of dolphin population size and areas of preferred habitat are needed to inform conservation management strategies^11,12^. Using aerial survey methods enabled us to produce large-scale abundance estimates and identify areas of preferred habitat across the gulfs (Spencer Gulf and Gulf St Vincent), and adjacent coastal and shelf waters in South Australia. This method enables an expansive view of the gulfs to inform the development of conservation and management measures in this region. The high abundance and density of bottlenose dolphins (likely *T. australis*) in the South Australian gulfs highlights the importance of these areas for this species in southern Australian waters.

Overall, abundance of bottlenose dolphins in the study area was similar between seasons, with estimates of around 3,200 and 3,500 bottlenose dolphins for summer/autumn and winter/spring, respectively. Abundance estimates for the four individual strata showed little differences between the two seasons, with higher overall abundance in gulf waters, lower abundance in protected shelf waters, and extremely low abundance in shelf waters adjacent to open ocean waters. The similarity in estimates across seasons, for both the four strata and overall, suggests that this species likely has a localized distribution without large-scale seasonal movements into and out of the study area. This corroborates existing genetic data that show a pattern of restricted dispersal and hierarchical metapopulation structure over larger parts of southern Australian coastal waters^16,23^.

Aerial survey line-transect distance sampling can potentially introduce several biases to abundance and density estimates. These include perception bias (animals missed due to observer error), availability bias (animals missed because they were not available, for example if they were underwater), visibility bias (animals are difficult to see from the air), and group size bias (error when group sizes are not estimated correctly due to their behaviour or size of the group)^45–47^. We used a double-observer platform and applied a Mark-Recapture Distance Sampling (MRDS) analysis to account for perception bias, but could not adjust for availability bias. Data to correct for availability bias were not available for bottlenose dolphins, which may have resulted in an underestimate of both abundance and density. Availability bias corrections are available for common dolphins (*Delphinus delphis*) in the area. In this species, only small corrections needed to be applied due to clear waters in the wider region and a good visibility from the air^44^. As common dolphins form larger schools and may display a different diving pattern to bottlenose dolphins, we did not incorporate data from this species as a proxy here. Visibility bias was also not accounted for, but it is expected to be low due to the good visibility in the region, which can occasionally deteriorate close to shore in shallow sandy seafloor areas. It is therefore possible that some individuals may have been missed close to shore, where bottlenose densities were generally observed to be high, but school sizes were relatively small. This could potentially have biased the abundance and density estimates downwards. Lastly, group size bias is likely negligible in our study because of the small school sizes for bottlenose dolphins typical of the study area, which make it easier to count them^45,46^. In combination, these biases could have led to an underestimate in bottlenose dolphin abundance and density in the study area, but is unlikely to have resulted in overestimates in any sub-region, overall or in the different seasons. In aerial surveys, animal speed is typically slow relative to observer speed, thus independent animal movement is unlikely to cause biases^48^. The length of our survey periods, however, may have introduced biases in estimates as animals could have moved among transects and regions. This can lead to inflated estimates^49^, but double counting in line-transect distance sampling is generally not considered a cause of bias if such counts correspond to different units of counting effort, as it is the case here^48^. This bias is therefore also likely to be negligible for our study.

While acknowledging that methodologies and resulting biases also need to be considered when making comparisons of dolphin densities across regions worldwide and using different survey platforms, overall results from our study suggest that South Australian bottlenose dolphin densities, especially in the two South Australian gulfs, were high compared to those of common bottlenose dolphins (*T. truncatus*) in other regions of the world, where densities ranged between 0.001–0.37 individuals/km^2^ ^50–52^. However, when comparing the South Australian estimates to inshore bottlenose dolphins (*T. aduncus*) in other regions of Australia (e.g. estimated densities of 0.19–1.78 dolphins/km^2^ ^53–55^), the estimates in South Australia are not unusually high. Furthermore, in Coffin Bay, South Australia (a small embayment of 263 km^2^ immediately west of the study area with ideal habitat) bottlenose dolphin (*T*. cf. *australis*) densities derived from boat surveys were also high (1.16 dolphins/km^2^)^27^.

Habitat modeling analyses revealed an association of bottlenose dolphins with shallow and coastal waters, especially in the gulfs, and highest overall predicted densities in upper gulf and coastal gulf waters. The upper gulf waters are characterized by a relatively flat seafloor topography, a high salinity level, no freshwater inflow and limited water exchange^56^. Bottlenose dolphins in the gulfs showed an opposing association to mean SSTs in the two seasons and preferred warmer waters in summer/autumn and cooler waters in winter/spring. This may be driven by the dolphins’ preference to coastal waters rather than the temperatures themselves. Mean SSTs are reversed in the gulfs in the two seasons, and coastal and upper gulf waters in summer/autumn show higher mean SSTs than other areas of the gulfs, while in winter/spring mean SSTs are cooler in coastal and upper gulf waters^56^. Preference to coastal waters in turn could be mediated by the distribution of prey and possibly protection from predators. Bottlenose dolphins in Spencer Gulf are known to feed mainly on octopus (Octopodidae), cuttlefish (Sepiidae), squid (Loliginidae), some crustaceans, and a range of different fish species including jacks (*Pseudocaranx* sp.), trevallies (*Trachurus* sp.), sardines (*Sardinops sagax*) and silverbellies (*Parequula melbournensis*)^57^. South Australia is also known for its large marine predators, including the great white shark *(Carcharodon carcharias)*, which is regularly sighted in both Spencer Gulf and Gulf St Vincent, and south of the gulfs^58^.

Predictive distribution maps derived from habitat modeling show a high density of dolphins in coastal regions of both South Australian gulfs year round (see Fig. 5). Gulf St Vincent had the highest estimated densities, suggesting that the gulf has large areas of suitable habitat for this species including metropolitan waters off Adelaide. Along Adelaide’s coastal waters, bottlenose dolphins show a high level of site fidelity^10,26^. A vessel based photo-identification mark-recapture study conducted in Adelaide’s coastal waters over a three year period, in an area of 195 km^2^ and up to 7 km from shore, estimated a total of 95–239 bottlenose dolphins that utilized this area^26^. Some of the dolphins were identified as year-round residents to Adelaide’s coastal waters, while others were considered seasonal residents and occasional visitors, likely using the wider region of Gulf St Vincent ^see^^26^. High densities of bottlenose dolphins were also found in northern and coastal waters of Spencer Gulf, with distributional maps showing suitable habitat particularly during winter/spring in north-western gulf waters, near the town of Whyalla where giant cuttlefish (*Sepia apama*) aggregations occur^59^. Bottlenose dolphins in the region feed on these cuttlefish^59^.

Bottlenose dolphins (*Tursiops* spp.) are globally found in coastal waters, but can also occur in offshore environments. Different ecotypes of bottlenose dolphins have been recognized worldwide, often showing inshore and offshore types, and inshore types are typically found either in coastal, embayment or estuarine waters^23,50,60–64^. In metropolitan coastal waters off Adelaide, Gulf St Vincent, bottlenose dolphins favor shallow nearshore environments and temperate reefs in summer, shallow nearshore environments in autumn, and deeper waters of the gulf further from shore in winter^10^. However, there is no general consensus which specific habitat features or oceanographic variables are associated with high bottlenose dolphin densities around the world^9,10,38,65,66^. The range of oceanographic parameters and habitat features that are associated with high dolphin densities in different regions around the world demonstrates the broad behavioural and adaptive plasticity that bottlenose dolphins are capable of, and these associations are likely specific for each region. This emphasizes the limitations of extrapolating abundance estimates and local habitat models to large geographical areas with changing habitats beyond a study region.

Marine habitats are highly variable, both temporally and spatially, and dolphin associations with habitat features and environmental parameters are often mediated by the distribution of their prey. An understanding of these local conditions is therefore central for identifying important habitat. Here we showed that protected northern and coastal gulf waters with flat seafloor topography were associated with high bottlenose dolphin densities. In contrast to gulf waters, the predictive distributional maps and the data from the line-transect survey showed low densities of bottlenose dolphins in protected shelf waters, and negligible numbers in open shelf waters. Shelf waters, particularly those unprotected and adjacent to open ocean waters are therefore likely to be unfavorable habitats for these coastal dolphins. Based on the results from habitat modeling, we predict that important areas for coastal bottlenose dolphins could also be located west of our study area, in waters off the western Eyre Peninsula. There, semi-protected bays are found along the coast, and several small islands provide protection from open shelf and ocean waters. An aerial survey in coastal and shelf waters off the western Eyre Peninsula, however, found mainly common dolphins (*Delphinus delphis*) in shelf waters, but no bottlenose dolphins, with the latter restricted to waters <12 km from shore^44,67^. To the east of our study area in South Australia, bottlenose dolphins occur around Cape Jervis and off Victor Harbor, and also in waters of the wider Coorong region^16,33^. Waters off the Coorong are exposed to unsheltered shelf and open ocean, and these waters are considered a less favorable habitat for bottlenose dolphins. Further systematic surveys close to shore are needed to the east and west of our study area to better understand the distribution and abundance of bottlenose dolphins along the South Australian coast and beyond.

Coastal dolphins worldwide are impacted by many different anthropogenic activities, and mitigating these impacts requires knowledge about dolphin distribution and abundance, and geographic overlap with threats^7^. Our results indicate that the two South Australian gulfs in central South Australia are an important habitat for bottlenose dolphins. The cumulative human induced impact on bottlenose dolphins in these gulfs is unknown, but according to expert elicitation bottlenose dolphins are among the species with highest vulnerability scores, highlighting the particular susceptibility of this species to anthropogenic threats^12^. For example, over the past decade in southern Australia, interactions of marine mammals with trawl, gillnet and purse-seine fisheries received increased public and political attention^11^. The majority of the marine mammal species considered at risk of fishery-interactions, however, were data deficient for abundance in the regions of interest, including bottlenose dolphins, and this previously made an assessment of population level threats difficult^11^. Although expert elicitation processes for marine mammal abundance can be beneficial in some scenarios^68–70^, estimates based on real data are always preferable for estimating dolphin abundance^50,51,53,71–73^. Computer modeling approaches to assess long-term viability of populations also requires abundance estimates^71,74–76^ and an understanding of population genetic structure ^see^^16,23^. Our study provided abundance estimates for two genetic populations of bottlenose dolphins in South Australia (Spencer Gulf and Gulf St Vincent ^see^^16^), that can be used for predictive population modeling and to inform conservation management.

The distribution of bottlenose dolphins along the southern Australian coast is non-homogenous and disjunct^11,15,16^. Waters off this coast have a complex and unique oceanography^56,77–79^, which likely is one of the main contributing factors for the non-homogenous distribution. The two large South Australian gulfs are inverse estuaries with limited water circulation and a lack of freshwater inflow^56^. As a result, the gulfs are particularly susceptible to human induced impacts such as climate change, habitat destruction and pollution, yet high densities of bottlenose dolphins are found in these waters. Within these gulfs, these areas of high dolphin density overlap with those areas of high use by human or those impacted by anthropogenic activities^12,28,57,80^. However, in most of these areas little or no protection is given to bottlenose dolphins.

Climate change was rated as one of the highest threats to dolphins in the area, particularly changes in temperature and storms, ocean warming, ocean acidification and increases in salinity^12^. Such changes may affect the distribution and abundance of dolphins via the loss of prey, potentially leading to food limitations, poor health of dolphins and spread of diseases that are linked to warmer waters, for example morbillivirus^30^. One such morbillivirus outbreak has resulted in the mortality of at least 41 bottlenose dolphins of the Gulf St Vincent population in 2013^30^. Epizootic outbreaks are density-dependent phenomena, and densities such as observed in Gulf St Vincent and Spencer Gulf may facilitate further outbreaks and expansions of epizootic events as water temperatures rise. Altogether, it is important to provide managers with baseline data of current abundance, distribution and density of bottlenose dolphins in central South Australian waters, so that these data can be used for impact level assessments, marine park planning, assessments of potential and known anthropogenic threats and as a baseline for future studies that assess long-term population trends.

Our study revealed that the two large South Australian gulfs (Gulf St Vincent and Spencer Gulf) are important habitats for bottlenose dolphins and that they are mainly associated with the northern and coastal sections of the gulfs. These waters are also where most anthropogenic activities occur and where climate change is likely to have the largest effect. More research is required to further understand potential threats to dolphins in the area, especially in a time of increased impacts by humans and potential effects of climate change on shallow coastal habitats. Prior to this study, there was a gap in knowledge on bottlenose dolphin abundance, density and distribution in central South Australia, a large area in which coastal bottlenose dolphins of the putative South Australian endemic species, *T. australis*, are regularly sighted. This information is now available to assist in assessing the overall conservation status of bottlenose dolphins in the region^16^.

## Methods

### Ethics approval

All data were collected under a research permit of the Department of Environment and Natural Resources, Government of South Australia (E25889-1/2). Ethics approval was granted by the Flinders University and Southern Adelaide Health Service Animal Welfare Committee, permit number E326. All methods were carried out in accordance with relevant guidelines and regulations.

### Data collection

Line-transect aerial surveys were conducted in an area of 42,437.8 km^2^ covering central South Australian waters over two seasons, the austral summer/autumn (March–June) and winter/spring (August-October) of 2011. The surveyed area was divided into four strata: stratum 1 ‘central South Australian continental shelf waters’; stratum 2 ‘Spencer Gulf’; stratum 3 ‘Gulf St Vincent’; and stratum 4 ‘Investigator Strait’ (Fig. 2, Table 1; bottlenose dolphins in stratum 2 and 3 belong to two distinct genetic populations)^16^. We used automated survey design algorithms^81^ implemented in the software program DISTANCE^82^ to design a systematic line-transect survey with regular line spacing within each survey stratum. Transect lines were placed perpendicular to shore, east-west and north-south, to sample across different water depths and habitat types. Homogeneous coverage probability in each stratum was achieved by using automated survey design algorithms implemented in software program DISTANCE to design a systematic random line-transect survey with regular line spacing within each survey block (7–8 km between transects). Such design randomly superimposes a systematic set of parallel lines onto the surveys region according to the spacing specified for the systematic sampler lines. The transects were the same for the summer/autumn and winter/spring surveys (Fig. 2). North-south transects in stratum 1 extended south over the Australian continental shelf out to the 100 m depth contour considering aircraft flying range, airport locations and fuel accessibility in the region.

Aerial surveys in all four strata were conducted from a twin-engine, high-winged six-seater Partenavia aircraft commonly used for cetacean aerial surveys, fitted with rounded windows in the middle seats (location of mid-seat observers) and flat windows in the rear seats (location of rear-seat observers). Transects were flown at a relatively low altitude of 500 ft (152.4 m) and a speed of 100 kt (185 km/h) to allow for accurate dolphin species identification. The pilot used a pressure altimeter, which was calibrated before each flight to ensure that the aircraft altitude measurements were correct. We limited survey flights to conditions of Beaufort sea state ≤3 (wind less than 15 kt). Dolphin detections while on transect were made in ‘passing mode’, which means that survey effort was ongoing and not suspended to circle back upon a sighting. Observers were trained to distinguish between bottlenose dolphins (*Tursiops* spp.) and common dolphins (*Delphinus delphis*) from the air at a distance, but different *Tursiops* species were not distinguished from the air^44^. In cases where species or school sizes were uncertain during passing mode, we suspended survey effort to circle the animals for species and school size determination. A school was defined as individuals that were within a 100 m radius of each other and travelling in the same direction^83,84^. Survey effort was then resumed when the aircraft reached the point on the transect line where effort was previously suspended.

We conducted all surveys with a double-observer platform and a team of six people: the pilot, a front-right survey leader, two mid-seat observers (right and left) and two rear-seat observers (right and left). Mid-seat and rear-seat observers were visually and acoustically isolated from each other while on effort using a non-transparent curtain and by wearing aviation headsets. A digital two-track voice recorder was connected to both intercoms to record sightings separately for mid-seat and rear-seat observers as they were called out. On each side of the plane, sightings of the mid-seat observers were therefore independently recorded (digitally marked) from the sightings of the rear-seat observers (digitally re-captured) using a double-observer mark-recapture setup. Rounded windows in the mid-seats allowed for a view to the trackline directly below the aircraft. Flat windows in the rear-seats allowed for a 65 degree declination angle, equivalent to 71 m out from the transect line. The mark-recapture setup was therefore only effective in distances between 71 m from the transect line out to the later estimated strip width of the survey. For each sighting abeam, observers recorded declination angles to sightings using inclinometers, species identification, group sizes and swimming direction of individuals. Survey effort data and sighting conditions including sea state, turbidity, cloud cover and glare were recorded by the survey leader at the start and end of each transects, at each sighting, and when conditions changed. The survey leader (visually and acoustically connected to the mid-seat observers) entered all effort, sighting conditions, and sighting data called by the mid-seat observers with time stamp signals of position from a GPS system using Cybertracker software (available at http://www.cybertracker.org/) sequence developed specifically for dolphin aerial surveys, uploaded to a Getac PS336 handheld computer. The rear-seat observer data was recorded onto the digital voice recorder during the survey for later transfer into the sightings database.

### Data analysis

#### Line-transect distance sampling

We compared sightings of bottlenose dolphins from mid-seat and rear-seat observers for coinciding timing, side of the aircraft, declination angle (distance from track line), group size and dolphin swim direction to identify duplicate sightings. For sightings that were identified as duplicates (marked and re-captured by the two tandem observers) but had slight differences in declination angle or group size, we used average values for each duplicate sighting. We identified duplicate sightings by reviewing each potential duplicate sighting of the mid-seat and rear-seat observers on each side of the aircraft. Identification of duplicate sightings was obvious in our study because individuals formed distinct clusters in manageable densities that were easily identified by the observers. In cases where individuals of a species occur in complex grouping patterns or higher densities, other methodologies may be more suitable and time effective to reliably identify duplicate sightings and reduce biases in the abundance estimates^46^.

We calculated the number of unique sightings by adding the sightings of platform 1 (mid-seat observer) to the sightings by platform 2 (rear-seat observer) and subtracting those sightings that were duplicated. We used the software DISTANCE version 7^82^ and R version 3.3.1 R Development Core^85^ to estimate bottlenose dolphin abundance, density, and expected school size for each stratum and overall, and for each combination of seasons (summer/autumn and winter/spring) separately. An exploratory analysis was undertaken for both survey seasons separately, to remove outlier sightings at long distances, assess the respective reduction in detection probability with perpendicular distance, and to decide for the most appropriate truncation distances (right truncation)^48,86^. We right truncated sighting data for summer/autumn surveys at a perpendicular distance of 570 m from the transect line, and at 660 m for winter/spring surveys to remove outlier sightings at long distances. Left truncation was set to a perpendicular distance of 71 m from the transect line (start of overlap between mid- and rear-seat observers) (Fig. 3).

We ran MRDS engine in DISTANCE 7 for double observer platforms with observer 1 being the mid-seat observers right and left (conceptually marking sightings), and observer 2 being the rear-seat observers right and left (conceptually recapturing sightings). A double-observer platform allows for estimating the probability of detection at zero distance, g(0), which is in contrast to conventional distance sampling methods where this probability is assumed to be 1^87^. We selected a point independence (IO configuration) MRDS model to estimate detection probability. This configuration in the MRDS engine is preferred for dolphin aerial surveys because detection probabilities of observers can become more positively correlated as distance increases. For example, this can occur when large schools of dolphins are more likely to be detected at large distances by both observers, even though both observers act independently^87^. The IO configuration is useful when objects (i.e. dolphins) are unlikely to have moved between detection by one and the other observer in response to the survey platform, which is typical for many aerial surveys ^see^^73^. MRDS models were fitted using both the hazard rate and half normal key functions available in the DS model component, and by systematically varying and adding scale parameters one by one (Beaufort sea state, cloud cover, turbidity, glare, airplane side and school size). We selected Generalized Linear Models (GLMs) for the MR model component with ‘distance’ as a covariate^82^. For both seasons (summer/autumn and winter/spring) abundance was estimated for each stratum and for the study area overall. Density estimates were derived for each stratum and overall incorporating the probability of detection and total transect length. The variance of the density estimates was calculated following Innes *et al*.^88^. We selected the best fitting model for each season based on lowest AIC, lowest Coefficient of Variance (CV) and by assessing goodness-of-fit in quantile-quantile plots (qq-plots), Kolmogorov-Smirnov tests and Cramer von Mises statistics as suggested by^89^. Models with the same AIC generally produced the same abundance and density estimates, with a few exceptions. Since no model is perfect and several models may be appropriate to describe the same data^89^, we used a range of model assessment criteria as described above for model selection. Among models with lowest AIC, we chose the model with the best goodness-of-fit parameters and confirmed that the estimates made biological sense^89^.

#### Habitat modelling

We fitted GAMs to the binned counts of dolphins from the aerial survey, separately for the summer/autumn and winter/spring surveys, to ascertain environmental conditions associated with variation in dolphin abundance. Counts of dolphins were binned into 5 km sections along survey transects. This bin size was chosen to reduce the predominance of bins with 0 counts and to approximate the resolution of the spatially interpolated, remotely-sensed environmental data. Environmental variables considered included: sea surface temperature (SST), bathymetry (depth of sea floor), bathymetric gradient (sea floor slope), distance to nearest land and distance to shelf edge (defined as the 500 m isobath). Additional remotely sensed environmental variables such as chlorophyll *a* concentration and sea surface height anomaly lacked sufficient resolution to adequately capture true variability in this relatively small region or were unreliable (biased) due to the proximity to the coastline. All environmental data were sourced from the Australian Antarctic Data Centre (http:://data.aad.gove.au/aadc) via the *raadtools* R package (https://github.com/AustralianAntarcticDivision/raadtools).

Although SST is highly variable in both time and space, initial models including daily resolution SST data failed to converge. We therefore chose to model spatial variability in SST averaged over the duration of the aerial survey period. We calculated the average SST separately for the summer/autumn and winter/spring surveys, thereby including a coarse level of SST temporal variability in the analysis. To account for zero-inflation in the dolphin counts, we compared GAMs fit with negative binomial, quasi-Poisson, zero-inflated Poisson and Tweedie (estimating the *p* parameter within the model) distributions. The Tweedie model residuals most closely approximated a Normal distribution and we therefore chose this approach for subsequent model selection.

To perform model selection over the set of environmental covariates, we relied on the built-in selection capability of the gam function in the mgcv R package^90,91^. This approach adds an extra penalty to each term, potentially penalising it to zero and removing it from the model. We present plots of the estimated smooth terms and spatial predictions of dolphin abundance from the reduced models separately for the summer/autumn and winter/spring surveys, and report the % deviance explained.

## Supplementary information

Supplementary Table S1a and S1b

## Data Availability

The datasets generated and/or analysed during this study are available from the corresponding author on reasonable request.
