# Supplementary material for: Abundance estimates and habitat preferences of bottlenose dolphins reveal the importance of two gulfs in South Australia
Source: Sci Rep. 2019 May 29;9:8044. doi: 10.1038/s41598-019-44310-3 (PMC6541621; doi:10.1038/s41598-019-44310-3)
Supplement: Supplementary file 1 — Supplementary Table S1a and S1b [file 41598_2019_44310_MOESM1_ESM.pdf]

SUPPLEMENTARY INFORMATION

Title: Abundance estimates and habitat preferences of bottlenose dolphins reveal the importance of two gulfs in South Australia

Authors: Kerstin Bilgmann*[1,2,3]; Guido J. Parra[1]; Lauren Holmes[1]; Katharina J. Peters[1,4,5]; Ian D. Jonsen[3]; Luciana M. Möller[1,2]

[1]Cetacean Ecology, Behaviour and Evolution Laboratory (CEBEL), College of Science and Engineering, Flinders University, GPO Box 2100, Adelaide, South Australia 5001, Australia

[2]Molecular Ecology Laboratory, College of Science and Engineering, Flinders University, GPO Box 2100, Adelaide, South Australia 5001, Australia

[3]Marine Vertebrate Conservation and Evolution Laboratory, Department of Biological Sciences, Faculty of Science and Engineering, Macquarie University, North Ryde, Sydney, NSW 2109, Australia

[4]Global Ecology, College of Science and Engineering, Flinders University, GPO Box 2100, Adelaide, South Australia 5001, Australia

[5]Coastal-Marine Research Group, School of Natural and Computational Sciences, Massey University, Auckland 0745, New Zealand

*Corresponding author

Supplementary Table S1a and S1b:

Summary statistics of the Mark-Recapture Distance Sampling (MRDS) analyses in DISTANCE 7, for A: the summer/autumn survey; and B: the winter/spring survey. The most suitable model is marked with a black frame: DS and MR model components were fitted by systematically adding covariates one by one to build more complex models. Choice of model with 'best fit' was based on lowest AIC, lowest %CV and by assessing quantile-quantile (q-q) plots, Kolmogorov-Smirnov tests, and Cramer von Mises statistics. AIC = Akaike Information Criterion; D = Density; N = Abundance estimate over the study area; CV = Coefficient of Variance; df = degrees of freedom.

Supplementary Table S1a:

Analyis Engine_Key Function_DS Model_MR Model_Truncations	Delta AIC	AIC	D	95% Confidence Interval		CV	N	%CV	df	95% Confidence Interval	
MRDS_Hr_MCDS_beaufort-cloudcover_MR_distance_LT71_RT570	0.00	1097	0.08	0.055	0.124	0.21	3493	20.70	101.53	2327	5244
MRDS_Hr_MCDS_beaufort-cloudcover_MR_distance_glare_LT71_RT570	0.00	1097	0.08	0.055	0.124	0.21	3493	20.70	101.53	2327	5244
MRDS_Hr_MCDS_beaufort-cloudcover-glare_MR_distance_LT71_RT570	1.28	1099	0.08	0.054	0.122	0.21	3448	20.57	101.65	2303	5163
MRDS_Hr_MCDS_beaufort-cloudcover-turbidity_MR_distance_LT71_RT570	1.96	1099	0.08	0.055	0.123	0.21	3485	20.70	101.22	2321	5232
MRDS_Hr_MCDS_beaufort_MR_distance_LT71_RT570	2.64	1100	0.08	0.053	0.121	0.21	3416	20.90	102.65	2267	5147
MRDS_Hr_MCDS_beaufort-cloudcover-side_MR_distance_LT71_RT570	3.89	1101	0.08	0.022	0.320	0.76	3522	76.42	78.61	913	13586
MRDS_Hr_MCDS_beaufort-glare_MR_distance_LT71_RT570	3.91	1101	0.08	0.053	0.120	0.21	3397	20.63	103.55	2266	5093
MRDS_Hr_MCDS_beaufort-trubidity_MR_distance_LT71_RT570	4.60	1102	0.08	0.053	0.121	0.21	3413	20.90	102.23	2265	5144
MRDS_Hn_MCDS_beaufort-cloudcover_MR_distance_LT71_RT570	5.81	1103	0.13	0.077	0.204	0.25	5316	25.01	118.84	3264	8658
MRDS_Hn_MCDS_beaufort-cloudcover_MR_distance_glare_LT71_RT570	5.81	1103	0.13	0.077	0.204	0.25	5316	25.01	118.84	3264	8658
MRDS_Hr_MCDS_beaufort-cloudcover-turbidity-side_MR_distance_LT71_RT570	5.86	1103	0.08	0.021	0.320	0.77	3509	76.64	77.46	907	13584
MRDS_Hr_MCDS_cloudcover_MR_distance_LT71_RT570	6.54	1104	0.09	0.059	0.139	0.22	3839	21.81	102.75	2504	5887
MRDS_Hr_MCDS_beaufort-side_MR_distance_LT71_RT570	6.54	1104	0.08	0.018	0.358	0.88	3383	87.56	77.96	753	15190
MRDS_Hn_MCDS_beaufort-cloudcover-glare_MR_distance_LT71_RT570	7.34	1105	0.13	0.077	0.202	0.25	5303	24.74	119.33	3273	8592
MRDS_Hn_MCDS_beaufort_MR_distance_LT71_RT570	7.46	1105	0.12	0.073	0.193	0.25	5053	24.86	123.29	3112	8205
MRDS_Hn_MCDS_cloudcover_MR_distance_LT71_RT570	7.56	1105	0.12	0.076	0.188	0.23	5091	23.05	110.95	3243	7991
MRDS_Hn_MCDS_beaufort-cloudcover-turbidity_MR_distance_LT71_RT570	7.77	1105	0.13	0.077	0.204	0.25	5300	25.12	118.14	3248	8650
MRDS_Hn_MCDS_beaufort-glare_MR_distance_LT71_RT570	8.22	1105	0.12	0.074	0.193	0.25	5067	24.58	125.05	3138	8183
MRDS_Hr_CDS_MR_distance_LT71_RT570	8.96	1106	0.09	0.055	0.133	0.22	3634	22.28	93.32	2347	5625

**Analyis Engine_Key Function_DS Model_MR Model_Truncations**	**Delta AIC**	**AIC**	**D**	**95% Confidence Interval**		**CV**	**N**	**%CV**	**df**	**95% Confidence Interval**	
MRDS_Hn_MCDS_beaufort-trubidity_MR_distance_LT71_RT570	9.46	1107	0.12	0.073	0.194	0.25	5055	24.90	122.45	3111	8214
MRDS_Hr_MCDS_glare_MR_distance_LT71_RT570	9.67	1107	0.09	0.056	0.131	0.22	3617	21.78	98.60	2360	5545
MRDS_Hn_MCDS_glare_MR_distance_LT71_RT570	10.64	1108	0.11	0.073	0.176	0.23	4802	22.69	109.28	3080	7487
MRDS_Hr_MCDS_turbidity_MR_distance_LT71_RT570	10.96	1108	0.09	0.055	0.133	0.22	3633	22.29	93.12	2346	5625
MRDS_Hn_MCDS_beaufort-side_MR_distance_LT71_RT570	11.33	1109	0.12	0.005	2.897	3.45	5088	344.61	74.33	211	122956
MRDS_Hn_CDS_MR_distance_LT71_RT570	11.34	1109	0.11	0.072	0.178	0.23	4808	22.95	98.62	3067	7537
MRDS_Hn_MCDS_beaufort-cloudcover-turbidity-side_MR_distance_LT71_RT570	11.76	1109	0.13	0.005	3.024	3.44	5314	343.94	72.33	220	128322
MRDS_Hr_MCDS_side_MR_distance_LT71_RT570	12.61	1110	0.09	0.011	0.754	1.47	3778	146.95	76.06	446	31985
MRDS_Hn_MCDS_turbidity_MR_distance_LT71_RT570	13.10	1110	0.11	0.072	0.177	0.23	4795	23.04	99.41	3053	7530
MRDS_Hn_MCDS_side_MR_distance_LT71_RT570	15.29	1113	0.11	0.004	3.178	3.93	4811	393.06	75.27	172	134864

Supplementary Table S1b:

Analyis Engine_Key Function_DS Model_MR Model_Truncations	Delta AIC	AIC	D	95% Confidence Interval		CV	N	%CV	df	95% Confidence Interval	
MRDS_Hn_MCDS_glare-cloudcover_MR_distance_LT71_RT660	0.00	1613	0.09	0.063	0.142	0.21	4006	20.60	83.48	2671	6009
MRDS_Hn_MCDS_glare-cloudcover_MR_distance_beaufort_LT71_RT660	0.00	1613	0.09	0.063	0.142	0.21	4006	20.60	83.48	2671	6009
MRDS_Hn_MCDS_glare-cloudcover_MR_distance_turbidity_LT71_RT660	0.00	1613	0.09	0.063	0.142	0.21	4006	20.60	83.48	2671	6009
MRDS_Hn_MCDS_glare-cloudcover_MR_distance_side_LT71_RT660	0.00	1613	0.09	0.063	0.142	0.21	4006	20.60	83.48	2671	6009
MRDS_Hr_MCDS_glare-cloudcover_MR_distance_LT71_RT660	0.06	1613	0.08	0.051	0.113	0.20	3213	20.36	75.77	2151	4801
MRDS_Hr_MCDS_glare-cloudcover_MR_distance_beaufort_LT71_RT660	0.06	1613	0.08	0.051	0.113	0.20	3213	20.36	75.77	2151	4801
MRDS_Hr_MCDS_glare-cloudcover_MR_distance_turbidity_LT71_RT660	0.06	1613	0.08	0.051	0.113	0.20	3213	20.36	75.77	2151	4801
MRDS_Hr_MCDS_glare-cloudcover_MR_distance_side_LT71_RT660	0.06	1613	0.08	0.051	0.113	0.20	3213	20.36	75.77	2151	4801
MRDS_Hr_MCDS_glare_MR_distance_LT71_RT660	0.09	1613	0.08	0.051	0.117	0.21	3276	21.10	75.22	2162	4964
MRDS_Hn_MCDS_glare-side_MR_distance_LT71_RT660	0.59	1614	0.10	0.062	0.150	0.22	2007	22.21	83.08	1325	3040
MRDS_Hn_MCDS_glare_MR_distance_LT71_RT660	1.22	1614	0.09	0.062	0.142	0.21	3987	20.90	72.86	2640	6020
MRDS_Hr_MCDS_glare-side_MR_distance_LT71_RT660	1.48	1615	0.08	0.050	0.114	0.21	3197	20.89	70.89	2117	4828
MRDS_Hr_MCDS_glare-turbidity_MR_distance_LT71_RT660	1.83	1615	0.08	0.051	0.118	0.21	3291	21.10	77.49	2172	4987
MRDS_Hr_MCDS_glare-beaufort_MR_distance_LT71_RT660	2.09	1615	0.08	0.051	0.117	0.21	3275	21.09	75.17	2161	4962
MRDS_Hn_MCDS_glare-turbidity_MR_distance_LT71_RT660	2.69	1616	0.09	0.062	0.141	0.21	3988	20.73	74.43	2650	6001
MRDS_Hn_MCDS_glare-beaufort_MR_distance_LT71_RT660	3.22	1616	0.09	0.062	0.142	0.21	3984	20.88	73.03	2640	6013
MRDS_Hr_MCDS_cloudcover_MR_distance_LT71_RT660	8.53	1622	0.07	0.048	0.106	0.20	3023	20.19	72.41	2029	4503
MRDS_Hn_CDS_MR_distance_LT71_RT660	8.61	1622	0.09	0.059	0.135	0.21	3803	20.80	69.06	2523	5734
MRDS_Hr_CDS_MR_distance_LT71_RT660	8.61	1622	0.09	0.059	0.135	0.21	3803	20.80	69.06	2523	5734
MRDS_Hn_MCDS_cloudcover_MR_distance_LT71_RT660	8.88	1622	0.09	0.060	0.134	0.20	3801	20.23	73.13	2550	5666

Analyis Engine_Key Function_DS Model_MR Model_Truncations	Delta AIC	AIC	D	95% Confidence Interval		CV	N	%CV	df	95% Confidence Interval	
MRDS_Hn_MCDS_side_MR_distance_LT71_RT660	9.36	1623	0.09	0.060	0.137	0.21	3842	20.86	71.32	2546	5799
MRDS_Hr_MCDS_side_MR_distance_LT71_RT660	10.09	1623	0.07	0.046	0.103	0.20	2927	20.45	66.46	1954	4385
MRDS_Hn_MCDS_beaufort_MR_distance_LT71_RT660	10.61	1624	0.09	0.059	0.135	0.21	3802	20.78	69.20	2523	5729
MRDS_Hn_MCDS_turbidity_MR_distance_LT71_RT660	10.61	1624	0.09	0.059	0.135	0.21	3801	20.89	70.32	2517	5740
MRDS_Hr_MCDS_turbidity_MR_distance_LT71_RT660	10.74	1624	0.07	0.049	0.111	0.21	3129	20.97	74.16	2070	4731
MRDS_Hr_MCDS_beaufort_MR_distance_LT71_RT660	11.00	1624	0.07	0.047	0.106	0.21	2992	20.59	68.58	1993	4492
